# Neural correlates of decision making after unfair treatment

**DOI:** 10.3389/fnhum.2015.00123

**Published:** 2015-03-05

**Authors:** Yan Wu, Yufeng Zang, Binke Yuan, Xuehong Tian

**Affiliations:** ^1^Department of Psychology, College of Education, Hangzhou Normal UniversityHangzhou, China; ^2^Zhejiang Key Laboratory for Research in Assessment of Cognitive Impairments, Hangzhou Normal UniversityHangzhou, China

**Keywords:** unfairness, ultimatum game, dictator game, anterior insula, ReHo

## Abstract

Empirical evidence indicates that people are inequity averse. However, it is unclear whether and how suffering unfairness impacts subsequent behavior. We investigated the consequences of unfair treatment in subsequent interactions with new interaction partners and the associated neural mechanisms. Participants were experimentally manipulated to experience fair or unfair treatment in the ultimatum game (UG), and subsequently, they were given the opportunity to retaliate in the dictator game (DG) in their interactions with players who had not played a role in the previous fair or unfair treatment. The results showed that participants dictated less money to unrelated partners after frequently receiving unfair offers in the previous UG (vs. frequently receiving fair offers in the previous UG), but only when they were first exposed to unfair UG/DG. Stronger activation in the right dorsal anterior insula was found during receiving unfair offers and during the subsequent offer-considering phase. The regional homogeneity (ReHo), a measure of the local synchronization of neighboring voxels in resting-state brain activity, in the left ventral anterior insula and left superior temporal pole was positively correlated with the behavior change. These findings suggest that unfair treatment may encourage a spread of unfairness, and that the anterior insula may be not only engaged in signaling social norm violations, but also recruited in guiding subsequent adaptive behaviors.

## Introduction

Humans are unique among all species in the extent to which they enforce social norms through the strategy of “an eye for an eye, a tooth for a tooth.” Fairness norms, in particular, are enforced by reciprocally fair behaviors. As a current influential economist, Matthew Rabin, noted, “People are willing to sacrifice their own material well-being to help those who are being kind, and are willing to sacrifice their own material well-being to punish those who are being unkind” (Rabin, 1993). We extended this thought by asking whether unfairness could spread to unrelated others. Although ample examples of retaliatory behavior against innocent people can be found in the real world, such as campus shootings and man-made bus fires, empirical studies are very rare in this field.

There is a broad consensus about the impulse to punish violators of social norms in studies in the field of economics, psychology and neuroscience. One useful way of assessing this behavior is the ultimatum game (UG), in which a proposer offers some portion of endowed money to a responder and the responder can either accept or reject the offer. Players either receive money if the responder accepts the offer or receive nothing if the responder chooses to decline the offer (Güth et al., 1982). Behaviorally, responders routinely reject substantial offers at a cost to themselves if the offers are unfair (Camerer and Thaler, 1995), irrespective of the stake size (Lisa, 1999; Munier and Zaharia, 2002), anonymity (Bolton and Zwick, 1995), the bargaining domain (Berger et al., 2012; Wright et al., 2012), or their cultural backgrounds (Henrich et al., 2006). Neuroscience studies using the UG showed that unfair offers were associated with increased activity in the anterior insula (Sanfey et al., 2003; Tabibnia et al., 2008) and that rejections of unfair offers were associated with reward-related brain regions such as the dorsal striatum (Osumi et al., 2010; Crockett et al., 2013; Wu et al., 2014). Previous findings have also suggested the involvement of the anterior cingulate cortex (ACC) (Kirk et al., 2011), ventromedial prefrontal cortex (VMPFC) (Koenigs and Tranel, 2007), and dorsal lateral prefrontal cortex (DLPFC) (Sanfey et al., 2003; Knoch et al., 2006) in fairness-related decision making. Although there is important work that examines the neural substrates of inequity aversion, such as the effects of emotion (Hollmann et al., 2011; Kirk et al., 2011; Harle et al., 2012; Crockett et al., 2013; Grecucci et al., 2013), intentionality (Guroŭlu et al., 2010), decisions for the self or on behalf of third parties (Civai et al., 2012; Corradi-Dell'acqua et al., 2013), competing with peers (Halko et al., 2009), social status (Hu et al., 2014), and age (Harle and Sanfey, 2012; Bailey et al., 2013), the consequences of unfair treatment aside from the rejection of unfair offers remain largely unknown.

Previous research has revealed that when observing an unfair partner receiving pain, males exhibited decreased activation in empathy-related areas (the bilateral anterior insula) and increased activation in reward-related areas (the nucleus accumbens) (Singer et al., 2006). These findings coincide with the economic model of social preference that people have an intrinsic taste for punishing others who violate social norms (Rabin, 1993; Fehr and Schmidt, 1999). Evidence is also observed in studies showing that victims are satisfied when they decide to punish or take revenge on offenders (Gollwitzer and Denzler, 2009). Areas known to be involved in reward processing, such as the ventral striatum (the nucleus accumbens) and the dorsal striatum (caudate), are activated when participants punish defectors' abuse of trust (de Quervain et al., 2004; Baumgartner et al., 2012) and selfish allocations (Strobel et al., 2011) and when participants reject unfair divisions (White et al., 2013). Beyond the rewarding hypothesis of punishing norm violations, recent findings of the increased periaqueductal gray (PAG) and inverse deactivation of VMPFC with higher punishments of unfairness are consistent with reactive aggression response, in which a punishing response may represent a reactive aggressive response to provocation (White et al., 2014). Typically, participants in these studies were allowed to retaliate against or administer punishment to related offenders. It is unclear whether and how individuals would take revenges on innocent others when they are hurt by social norm violations.

The present study offers a new insight into the behavioral and brain responses associated with decision change after fair and unfair treatment. To emphasize how participants adapt their behaviors toward unrelated others based on previous experience, rather than on their responses to related offenders, we asked participants to first act as responders in the ultimatum game and then to act as dictators in the dictator game (DG, which is the same as the UG, except that the responder's only choice is to accept offers). A critical manipulation was that the offers in the two UG blocks could be mainly fair (83% fair offers) or mainly unfair (83% unfair offers). Importantly, the partners in the two DG blocks were completely unrelated and were not responsible for the UG offers. In this way, we were able to test whether the participants' allocation in the DG would be affected by their previous fair or unfair treatment in the UG and how these behavioral differences related to the brain.

A similar UG design has been used in a previous study but with different aims (Xiang et al., 2013). In their study, two groups of participants were randomly assigned to receive high offers ($12 ± $1.5, out of $20) or low offers ($4 ± $1.5, out of $20) in the first 30 trials and then to receive medium offers ($8 ± $1.5, out of $20) in the last 30 trials. Another two groups of participants were randomly assigned to receive offers in the medium-high or medium-low order. The researchers used this norm-training task and a Bayesian observer model to track the neural correlates of norm prediction errors. They found that group high-medium participants preadapted to 30 high offers rejected medium offers more frequently than group low-medium participants who preadapted to 30 low offers, and the activity of ventral striatum and anterior insula correlated with both positive and negative norm prediction errors. This study contributes to the understanding of the underlying neural and behavioral responses of social norm violations. However, it remains unclear whether social norm violations impact upon future behavior toward others.

In the present study, of crucial interest were the behavioral and neural differences between the DG blocks after fair vs. unfair treatment. Since human behavior is adaptive to social context (Xiang et al., 2013), we predicted a behavior change in the DG following unfair vs. fair treatment. Specifically, participants may be tempted to retaliate against unrelated others and vent on innocent partners all of the spite they feel against their previous unfair partners, and they should dictate less in the DG following unfair treatment in the UG than they do in the DG following fair treatment in the UG. Neutrally, we predicted that brain regions involved in processing social norm violations would be activated during exposure to unfair offers and during subsequent decision stage, including bilateral anterior insula (Xiang et al., 2013; White et al., 2014). We hypothesized that the behavior change was mainly driven by punishment impulse akin to reactive aggression, and predicted a differential response after unfair treatment in punishment-related areas, such as periaqueductal gray and VMPFC (White et al., 2014). As previous research revealed a strong activation in the ACC and increased posterior cingulate cortex (PCC) activity in fair as compared to unfair offers in the dictator game (Weiland et al., 2012), we predicted that these areas were also survived in the contrast between fair DG and unfair DG.

To elucidate the inter-individual variations in behavioral differences, we recorded baseline brain activity using resting state functional magnetic resonance imaging (RS-fMRI). Regional homogeneity (ReHo) measures the temporal synchronization of the time series of an area's nearest neighbors (Zang et al., 2004). This measure is based on the hypothesis that clusters of voxels rather than single voxels manifest intrinsic brain activity. ReHo requires no a priori definition of ROIs and can provide information about the local or regional activity of regions throughout the brain. As an index of baseline brain activity, ReHo has been widely used in the resting-state literature. In healthy subjects, ReHo measures have been proven to be an effective tool for investigating the neural basis of individual differences in behavior (Tian et al., 2012), personality (Wei et al., 2011), and intelligence (Wang et al., 2011). In the present study, we used the ReHo-behavior correlations to explore how the individual variability in the local connectivity in the baseline brain activity associated with their decision behavior. Significant ReHo-behavior correlations demonstrated that a higher (positive correlation) or lower (negative correlation) regional synchronization of certain areas correlates with larger behavior change between the DG blocks (e.g., more likely to be affected by the previous unfair treatment). Because of the lack of existing literature, this part of the study was exploratory, and no specific hypothesis could be made for the results.

## Methods

### Participants

Thirty-two undergraduate and graduate students took part in the experiment (eight males/24 females; mean age = 22.31 years, SD = 2.35). All participants were healthy right-handed volunteers without neurological or psychiatric impairments. Participants were paid 100 RMB (equivalent to US$16) each per hour for participation plus a bonus based on their decisions in the UG and DG. All participants gave written informed consent for participation in the experiment and were informed of their right to discontinue participation at any time. Participants were naïve to the purpose of the experiment. The study was approved by the ethics committee of the Center for Cognitive and Brain Disorders, Hangzhou Normal University.

### Task and procedure

Participants were first required to undergo an 8-min resting-state fMRI scan before the decision-making task based on the consideration that RS-fMRI signals could possibly be contaminated by preceding events. They then received written and oral instructions about the UG and DG game rules in the scanner (without training before the scans). The following information was emphasized to the participants both in writing and orally: (1) their partners were real students from the same university who would not be shown immediately; (2) offers in the UG task were collected from real students in advance; (3) partners would vary in each round; and (4) both the participants and their partners would be paid real money (one round from each block would be chosen at random by the end of the experiment).

Cover stories are always a challenge in social decision-making research that employs game paradigms. The offers in the UG were usually predetermined, but participants were told that offers were real and had been made by other participants who had participated previously (Boksem and De Cremer, 2010) or by partners via the Internet (Stephen and Pham, 2008). In the present study, participants were shown a list of their partners' names at the end of the instruction, which was used to enhance the plausibility of the cover story. The names were divided into four groups corresponding to four experimental blocks. Each name corresponded to each round of the UG (two block, 36 rounds per block) and the DG (two block, 30 rounds per block), for a total of 132 names. The names for the UG were taken from preceding participants who had taken part in the pilot study. The names for the DG were collected from freshmen with whom the participants were unfamiliar. The purposes of showing the names were (a) to enhance the plausibility of the experimental setup, (b) to implement a repeated one-shot ultimatum game and dictator game, and (c) to promote the participants' personal involvement in each trial. To eliminate the confounding effect of social distance on decision making (Charness and Gneezy, 2008), the names were replaced by codes in the experimental blocks.

There were four experimental blocks—(1) a fair UG block, (2) a DG block following a fair UG, (3) an unfair UG block, and (4) a DG block following an unfair UG—following a detailed instruction. Unknown to each participant, the offers in the UG were manipulated by the experimenter. In the fair UG block, participants played as the responder and received a randomized series of 36 offers (15 repetitions for offers of 5/5 and 4/6; two repetitions for offers of 3/7, 2/8, and 1/9; the number in front of the slash indicated the amount proposed for the participant, and the number following the slash indicated the amount left to the proposer; the sum was RMB10 for each round). In the unfair UG block, 15 repetitions were made for offers of 1/9 and 2/8, and two repetitions for offers of 3/7, 4/6, and 5/5. A DG block of 30 trials followed the fair UG block, and a DG block of 30 trials followed the unfair UG block. The order of the fair and unfair UG blocks (and thus the subsequent DG blocks) was counterbalanced between participants. The only difference between the DG blocks was whether the previous UG offers had been mainly fair or unfair. The difference between the UG and the DG was highlighted in the written instruction and was emphasized orally by the experimenter.

**Figure 1:**
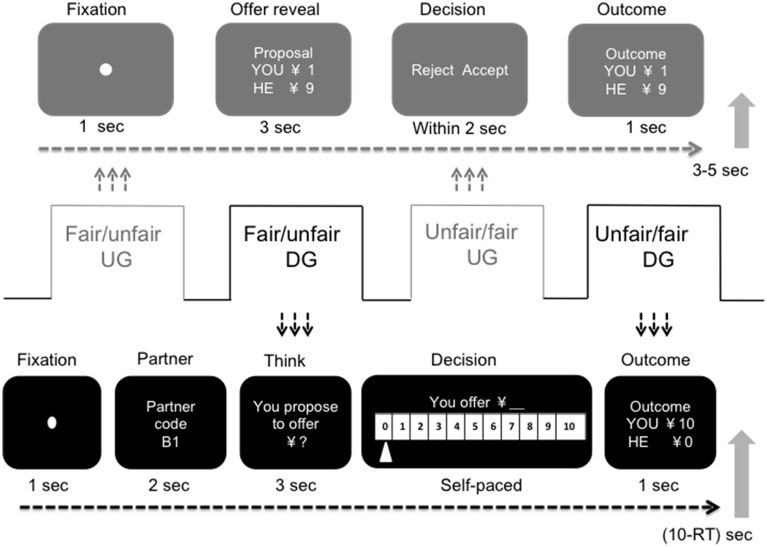
**Schematic display of the experimental paradigm and design of a single trial in the ultimatum game and the dictator game.** The experimental session consisted of four blocks, two for the ultimatum game, with 36 trials in each block, and two for the dictator game, with 30 trials in each. For the ultimatum game block, the participants played the role of responder and were asked to make a decision on previously collected proposals. For the dictator game block, the participants played the role of dictator and were asked to make a decision on how much they wanted to offer. In the fair UG, 30 of the 36 trials were fair offers (5/5 or 4/6); in the unfair UG, 30 of the 36 trials were unfair offers (1/9 or 2/8). In the fair DG, the DG block followed fair treatment in the UG block, and in the unfair DG, the DG block followed unfair treatment in the UG block. The order of the fair and unfair UG blocks was counterbalanced between participants.

The experimental display is presented in Figure 1. In the UG, a fixation picture (duration 500 ms) indicated the beginning of a new round. The participant then saw the offer. After 3 s, the response screen reminded the participant to make a decision by pushing the corresponding button using either the right index or middle finger. Participants were asked to respond within 2 s to keep them alert (given the sleep-inducing environment in the scanner especially for repeating task). If they failed to respond within 2 s, a confirmation screen texted “response failed” displayed and reminded them that they would get zero for the current round and the partner would get the proposed amount (which had been made clear in the instruction). Immediately after they pressed the button, the outcome for that round was presented for 1000 ms. A randomized break period of 3–5 s followed. A block of 30 DG trials followed the UG. In the DG, each participant first saw a fixation picture for 500 ms that indicated the beginning of a new round. Then, the partner's code was shown for 2000 ms. The offer-considering screen cued the participant to consider how much he or she wanted to offer. The cue screen lasted for 3000 ms and was followed by a response screen that reminded the participant to make an offer by pushing the corresponding button to move left or right to the specific number (the default number could be zero or ten, counterbalanced between participants). The participants had a self-paced amount of time to make a decision, but they were asked to respond quickly. The outcome for that trial was presented for 1000 ms. A duration of 10 s minus the response time period separated the outcome from the beginning of the next round.

At the end, participants were debriefed and asked whether they had had doubts about the identity of their partners (i.e., “Did you ever doubt that your partners were real?”). However, none of them indicated this.

### fMRI data acquisition

fMRI data were obtained using a 3.0 Tesla GE Discovery MR-750 scanner at the Center for Cognitive and Brain Disorders, Hangzhou Normal University. Each participant underwent an 8-min fMRI scan during a conscious resting state immediately after the acquisition of the localizer images. Participants were instructed to simply keep their eyes closed, think of nothing in particular, and not fall asleep.

Both resting state and task fMRI data were gathered with a high-field, high-resolution head coil optimized for functional imaging. Functional T2^*^ images were acquired axially using an echo-planar imaging sequence that was sensitive to blood oxygen level-dependent (BOLD) contrast. The acquisition parameters were as follows: 37 slices, 2000/30 ms (TR/TE), 3.4/0 mm (thickness/gap), 220 × 220 mm (FOV), 64 × 64 (resolution), and 60° (flip angle). T1-weighted images covering the whole brain were then obtained sagittally with the following parameters: 180 slices (to achieve 176 slices, with two slices at each end being discarded), 8100/3.1 ms (TR/TE), 1.0/0 mm (thickness/gap), 250 × 250 (resolution), 250 × 250 mm (FOV), 8° (flip angle), 1 × 1 × 1 mm (voxel size, isotropic), 450 (T1, preparation time), and 31.25 kHz (bandwidth).

### Resting state fMRI data preprocessing

For the resting state fMRI data, the first 10 volumes were discarded to ameliorate the possible effects of scanner instability and subjects' adaptation to the environment. The remaining functional scans were corrected for within-scan acquisition time differences between slices and then realigned to the first volume to correct for within-run head motions. This realigning step provided a record of head motions within each fMRI run. Each functional volume was registered to the participant's anatomical image and was then spatially normalized to the East Asian brain template provided by SPM8 (http://www.fil.ion.ucl.ac.uk/spm/software/spm8/) and resampled to 3 × 3 × 3 mm^3^ to the standard Montreal Neurologic Institute (MNI) template brain. The functional scans then underwent linear regression, and the influences of the linear trends were subsequently removed from the data. Finally, the waveform of each voxel was temporally band-pass filtered (0.01–0.1 Hz) to reduce the influences of low-frequency drift and high-frequency noise. The data preprocessing and statistical analysis were performed using Data Processing Assistant for Resting-State fMRI (DPARSF) (Yan and Zang, 2010), which is a pipeline data processing toolbox that integrates the preprocessing modules of SPM8 and the post-processing modules of REST software (www.restfmri.net) (Song et al., 2011).

### ReHo analysis

ReHo valuates the similarity or synchronization between the time series of a given voxel and its nearest neighbors (Zang et al., 2004). ReHo was performed on a voxel-by-voxel basis by calculating the Kendall's coefficient of the time series concordance of a given cluster of neighboring voxels (Zang et al., 2004). Here, cubic clusters of 27 voxels were used, and the ReHo-value of each cubic cluster was assigned to the central voxel. A larger ReHo-value for a given voxel indicated a higher local synchronization of RS-fMRI signals among neighboring voxels. To minimize the whole-brain effect, voxel ReHo-values were scaled by dividing each participant's value by the mean value of his or her whole-brain ReHo. The mean ReHo images were then spatially smoothed using a Gaussian kernel of FWHM 8.0 mm. All of these procedures were performed using the REST software and SPM8. Four participants were not included in further RS-FMRI analysis, one because of head motions greater than a 3-mm deviation in the center of mass in the x-, y-, or z-dimensions and the other three because of the poor co-registration of their anatomical and functional images.

### ReHo-behavioral correlation analysis

Pearson's correlation analysis between the mean ReHo-values and the behavior change was performed in a voxel-wise manner. Each participant's behavior change was calculated by the difference between the mean offers in the two DG blocks. To control for Type I error, a corrected significance level of *p* < 0.05 was obtained using AlphaSim with cluster size >389 mm^3^ and an individual voxel height threshold of *p* < 0.05. The ReHo-behavioral correlation map was finally superimposed onto a template that was provided in MRICRO software (http://www.mccauslandcenter.sc.edu/mricro/) for display; all significant correlations were presented in MNI coordinates.

### Task fMRI data analysis

The task fMRI data analysis was conducted on the UG and DG data. Preprocessing of the functional data was performed with DPARSF software. The data were realigned to the first volume to minimize the effects of head movements on the data analysis. Anatomical and functional images were co-registered and normalized to the East Asian brain template provided by SPM8. The data were then smoothed with a Gaussian kernel of FWHM 8.0 mm. Five participants were excluded, leaving 27 participants in the final analysis; one was excluded because of excessive head motions (greater than a 3-mm deviation in the center of mass in the x-, y-, or z-dimensions) and four because of the poor co-registration of their anatomical and functional images.

For the UG data analysis, we separately modeled the offer presentation, decision, and outcome in the fair and unfair UG block with delta functions convolved with a canonical hemodynamic response function. The offer presentation corresponding to each type of offers was modeled in separate regressors. As the main focus of the present study was the decision change in the DG, the decision phase in the UG was a regressor of no interest, and it was included to partial out variance due to changes associated with motor response. The six rigid body parameters were also included to account for head motion artifact. For each participant, contrasts were calculated between regression coefficients for fair (5/5 and 4/6) and unfair offers (1/9 and 2/8) at every voxel in the brain. Whole-brain analysis was performed by one-sample *t*-test that was used to determine where the average contrast value for the group as a whole (*n* = 27 differed significantly from zero (a random-effects analysis). Statistical maps were thresholded for significance (*p* < 0.001, uncorrected) and cluster size (≥ 10 voxels).

For the DG data analysis, a separate general linear model (GLM) was defined for each participant to examine the different neural responses to DG following fair and unfair treatment. We defined six regressors, with three events (offer-considering, offer-making, outcome) for each block (DG block following fair and unfair treatment). Additional regressors were included to model events that were of no interest (the onset of the fixation) and the six covariates per session that contained the realignment parameters that captured the subjects' movements during the experiment. Each regressor was convolved with a standardized model of the hemodynamic response.

For each participant, direct contrasts of parameter estimates for each event (offer-considering, offer-making, outcome) between the DG following fair treatment and the DG following unfair treatment were computed at each voxel of the brain. Whole-brain analysis used one-sample *t*-tests to identify voxels where the average contrast (DG following fair vs. unfair treatment) for the whole group (*n* = 27 participants) differed significantly from zero (i.e., a random effect analysis). The resulting map of the *t* statistic was thresholded at *p* < 0.001, uncorrected, with a spatial extent threshold of 10 contiguous voxels.

For the group analysis, a random-effects model was used with a small-volume correction for multiple comparison within the a priori regions of interest in the (1) bilateral anterior insula—8 mm sphere at MNI coordinates −34, 15, 2 and 36, 15, 3 (Sanfey et al., 2003), (2) periaqueductal gray with 8 mm sphere at the MNI coordinates −13.5, −22.5, 8.5 (White et al., 2014), (3) VMPFC with 8 mm sphere at the MNI coordinates 4.5, 46.5, −0.05 (White et al., 2014), (4) right ACC with 8 mm sphere at the MNI coordinates 5, 31, 39 (Weiland et al., 2012), and (5) right PCC with 8 mm sphere at the MNI coordinates 8, −29, 30 (Weiland et al., 2012). ROI analysis was performed by extracting beta-values centered on the independently defined coordinates derived from previous study.

## Results

### Behavioral results

**Figure 2:**
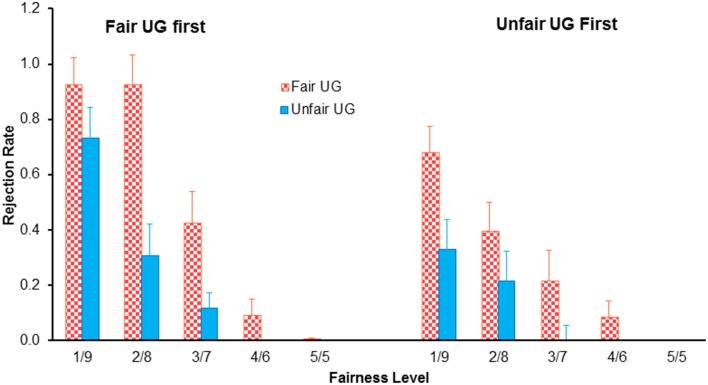
**The reject rate as a function of type of offers, experimental block, and the order of block in the ultimatum game.** Bars indicate standard error.

Five participants were excluded because of problems with their imaging data. For the remaining 27 participants, a two (block: fair UG vs. unfair UG) by five (type of offers: 5/5, 4/6, 3/7, 2/8, 1/9) by two (order of blocks: fair UG first vs. unfair UG first) mixed design repeated-measure ANOVAs of the rejection rate revealed a main effect of block, with higher rejection rate in fair UG (37%) than in unfair UG (32%), *F*_(1, 25)_ = 62.341, *p* < 0.001, η^2^_*p*_ = 0.714, a main effect of type of offers, with decreased rejection rate with increased fairness, *F*_(4, 100)_ = 55.796, *p* < 0.001, η^2^_*p*_ = 0.691, a main effect of order of blocks, with higher rejection rate for fair block first (35%) than that for unfair block first (19%), *F*_(1, 25)_ = 5.346, *p* = 0.029, η^2^_*p*_ = 0.176, and importantly, a significant three-way interaction effect of experimental block, offers, and order of blocks, *F*_(4, 100)_ = 3.251, *p* = 0.036, η^2^_*p*_ = 0.115 (Figure 2). When unfair block was first presented, the two (block: fair UG vs. unfair UG) by five (type of offers: 5/5, 4/6, 3/7, 2/8, 1/9) repeated-measure ANOVAs of the rejection rate revealed a main effect of block, with higher rejection rate in the fair UG (27%) than in the unfair UG (11%), *F*_(1, 13)_ = 21.958, *p* < 0.001, η^2^_*p*_ = 0.628, and a main effect of type of offers, with decreased rejection rate with increased fairness, *F*_(4, 52)_ = 14.037, *p* < 0.001, η^2^_*p*_ = 0.519, but not interaction effect of block and offers, *F*_(4, 52)_ = 2.554, *p* = 0.091, η^2^_*p*_ = 0.164. When fair block was first presented, the two by five repeated-measure ANOVAs revealed a main effect of block, with higher rejection rate in the fair UG (47%) than in the unfair UG (23%), *F*_(1, 12)_ = 41.129, *p* < 0.001, η^2^_*p*_ = 0.774, a main effect of type of offers, with decreased rejection rate with increased fairness, *F*_(4, 48)_ = 50.225, *p* < 0.001, η^2^_*p*_ = 0.807, and an interaction effect of block and offers, *F*_(4, 48)_ = 6.939, *p* = 0.004, η^2^_*p*_ = 0.366. *Post-hoc* analysis revealed a main effect of block for 1/9 offers (92% for fair UG vs. 73% for unfair UG, *F*_(1, 12)_ = 5.889, *p* = 0.032, η^2^_*p*_ = 0.329), 2/8 offers (92 vs. 31%, *F*_(1, 12)_ = 22.925, *p* < 0.001, η^2^_*p*_ = 0.838), 3/7 offers (42 vs. 12%, *F*_(1, 12)_ = 6.508, *p* = 0.025, η^2^_*p*_ = 0.352), but not for 4/6 offers and 5/5 offers (both approach to zero rejection).

**Figure 3:**
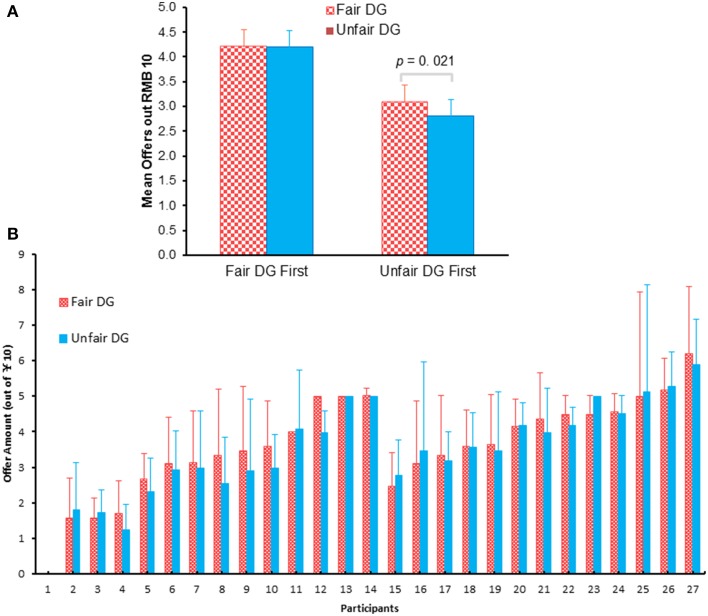
**The grand mean offers (A) and mean offers made by each participant (B) during the fair (following the fair UG) and unfair (following the unfair UG) DG blocks.** The absence of standard error bars indicates that the participant made the same offer in each trial. Participants 1–14 were first exposed to unfair UG/DG and then fair UG/DG. Participants 15–27 were opposite.

A two (block: fair DG vs. unfair DG) by two (order of the blocks: fair UG/DG first vs. unfair UG/DG first) mixed design repeated-measure ANOVAs of the average offer made by each participant in two DG blocks showed a main effect of fair treatment, *F*_(1, 25)_ = 4.466, *p* = 0.049, η^2^_*p*_ = 0.152, with fewer allocations following unfair UG (3.48 ± 1.37) than that following fair UG (3.62 ± 1.37), and a main effect of the order of blocks, *F*_(1, 25)_ = 7.046, *p* = 0.014, η^2^_*p*_ = 0.22, with fewer mean offers when unfair UG block was first presented (2.95) than that when fair UG block was first presented (4.20). The interaction effect of block and the order of block approached marginal significance, *F*_(1, 25)_ = 4.139, *p* = 0.053, η^2^_*p*_ = 0.142 (Figure 3A). When fair DG was first presented, there was no difference between offers in the fair DG and offers in the unfair DG, *F*_(1, 12)_ = 0.004, *p* = 0.948. However, when the unfair DG was first presented, there were more offers in the fair DG (3.08) than offers in the unfair DG (2.81), *F*_(1, 13)_ = 6.89, *p* = 0.021, η^2^_*p*_ = 0.346.

The offers participants made in the DG following the fair UG and in the DG following the unfair UG were showed for each participant (Figure 3B). It should be noted that not all participants (but most of them, i.e., 16 of 27) made less offers in the DG following the unfair UG than they made following the fair UG. Eight participants showed the opposite pattern, and another three showed no difference between the two blocks.

There was no significant difference between male and female participants [*t*_(26)_ = 0.22 *p* > 0.05] and no difference in the response time between the two DG blocks [2290 vs. 2247 ms, *F*_(1, 26)_ = 0.26; *p* > 0.05].

### Task fMRI results

**Figure 4:**
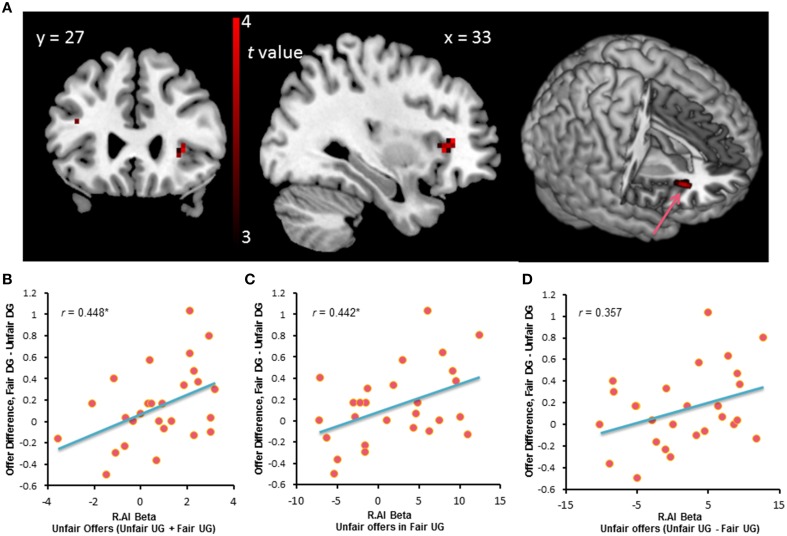
**(A)** Brain activation for the contrast between the unfair offers vs. with fair offers in UG. **(B,C)** The beta-value of the right dorsal anterior insula during unfair offers in fair and unfair UG positively correlated with behavior change in DG. ^*^p < 0.05 (two-tailed). **(D)** There was a trend that the behavior change in DG correlated with the differential activation in the right dorsal anterior insula during unfair offers between the fair UG and the unfair UG.

Whole-brain analysis on the UG data only found the right dorsal anterior insula that survived the contrast between fair and unfair offers, with the threshold of *p* < 0.001, uncorrected, *k* > 10. Compared with fair offers in the UG, unfair offers elicited activation in the right dorsal anterior insula (20 voxels, Brodmann Area 47, peak voxel at MNI coordinates 33, 27, 3, Figure 4A). We extracted the beta estimates in the right dorsal anterior insula corresponding to unfair offers in the fair and unfair UG and subjected these values to a correlation analysis with the offer change in the DG. Results showed that activation in the right dorsal anterior insula during unfair offers in UG was positively correlated with the behavior change in DG, *r* = 0.448, *p* = 0.019 (Figures 4B,C). And there was a trend that differential activation in the right dorsal anterior insula during unfair offers between the fair UG and the unfair UG was correlated with the behavior change in DG, *r* = 0.357, *p* = 0.068 (Figure 4D).

**Figure 5:**
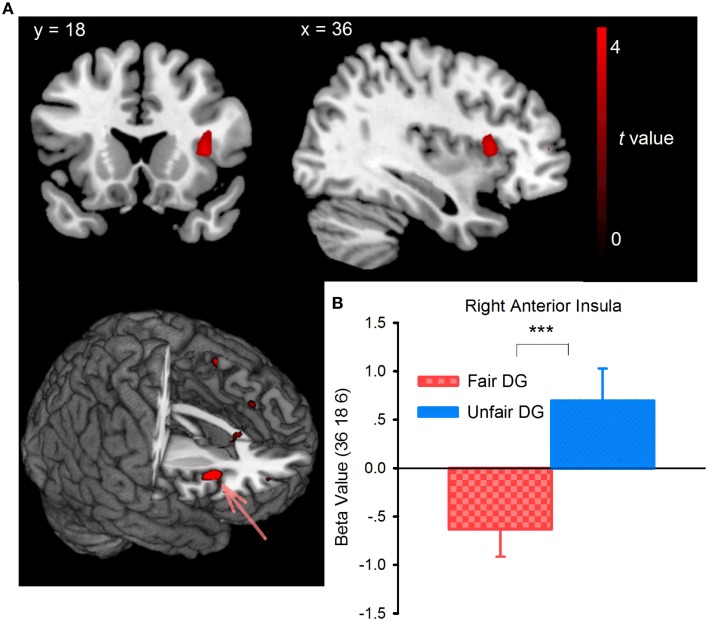
**Brain activity for the contrast between the fair (following the fair UG) and unfair (following the unfair UG) DG trials during the offer-considering phase (A) and (B) the beta-values extracted from the activation maximum within an 8-mm sphere around the right anterior insula ROI, whose coordinates were derived from previous studies (Sanfey et al., 2003)**. ^***^p < 0.001 (two-tailed).

Whole-brain analysis on the DG data found that during the offer-considering phase, the unfair DG (following the unfair UG) elicited activation in the right dorsal anterior insula (BA48, peak voxel at MNI coordinates 36, 18, 9, Figure 5A, Table 1), compared with the fair DG (following the fair UG). Small-volume correction (SVC) was performed over an 8-mm sphere around the coordinates identified from previous studies on inequity aversion (Sanfey et al., 2003). Confirming the pattern observed in the whole-brain analysis, the ROI-based analysis showed that the right dorsal anterior insula [peak MNI coordinates: 36, 18, 6; *p*FWE (SVC) = 0.007, *k* = 6] showed enhanced activity in the unfair DG relative to the fair DG. The beta estimates extracted from the peak activation voxel identified within the ROI of right dorsal anterior insula were plotted (Figure 5B). As the behavior results of DG suggested the effect of the order of block, we conducted a two (fair DG vs. unfair DG) by two (fair DG first vs. unfair DG first) mixed-design repeated-measures ANOVAs with the beta-value extracted from right dorsal anterior insula as dependent variable. The results revealed a main effect of fair treatment, *F*_(1, 25)_ = 17.207, *p* < 0.001, η^2^_*p*_ = 0.408, with positive activity in unfair DG (0.757) and negative activity in fair DG (−0.622). The order of block [*F*_(1, 25)_ = 0.81, *p* = 0.377, η^2^_*p*_ = 0.031], and the interaction between treatment and the order of block [*F*_(1, 25)_ = 0.05, *p* = 0.825, η^2^_*p*_ = 0.002] were not significant. There were no other differences in brain activation at the reported threshold for the whole brain analysis and for the ROI analysis of periaqueductal gray, VMPFC, ACC, and PCC. The reversed contrast (fair DG vs. unfair DG) revealed no supra-threshold activation.

**Table 1:** **Significant activation for the contrasts of the DG following fair vs. unfair treatment**.

**Regions**	**Hemisphere**	**MNI coordinates**			**Max *T*-value**	**Voxel size**
		***x***	***y***	***z***		
**OFFER-CONSIDERING PHASE (DG FOLLOWING THE UNFAIR UG ≫ DG FOLLOWING THE FAIR UG)**						
Anterior insula	R	36	18	9	4.22	11
**OFFER-MAKING PHASE (DG FOLLOWING THE FAIR UG ≫ DG FOLLOWING THE UNFAIR UG)**						
Middle temporal gyrus	R	42	−66	9	4.73	44
Middle occipital gyrus	R	36	−75	12	4.32	
Posterior insula	R	42	−9	−6	3.93	10
**OUTCOME PHASE (DG FOLLOWING THE FAIR UG ≫ DG FOLLOWING THE UNFAIR UG)**						
Post-central	R	42	−24	60	4.01	14
Middle occipital gyrus	L	−63	−12	−9	3.93	10

### ReHo-behavioral correlations

**Figure 6:**
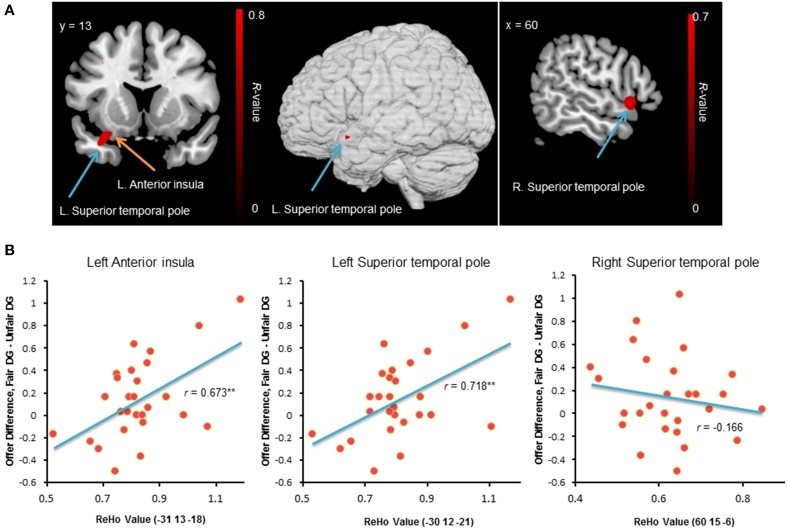
**Brain regions exhibited significant positive ReHo-behavioral correlations (A).** An *R*-value scale is shown. The numbers above each image refer to the y-coordinates of the Montreal Neurological Institute (MNI). The threshold was set at *p* < 0.05 (corrected). The lower panel **(B)** shows the correlation between behavior change and mean ReHo-value in the left ventral anterior insula and the bilateral superior temporal pole. ^**^p < 0.01 (two-tailed).

We employed ReHo of resting-state fMRI signals to investigate the functional basis of individual differences in behavior change in the DG. Unlike the task activation method that relies on the contrast between conditions, the ReHo measure is data-driven and focuses on the local synchronization of voxels within a specific brain area. At a threshold of *p* < 0.05 (corrected), significant positive ReHo-behavioral correlations were found in three clusters: (1) 51 voxels, Brodmann area 47, 38, peak MNI coordinates: −33, 12, −24, regions: the left superior temporal pole and the left ventral anterior insula (Figure 6A); (2) 37 voxels, Brodmann area 22, peak MNI coordinates: 60, 15, −6, region: the right superior temporal pole (Figure 6A); and (3) 26 voxels, Brodmann area 38, peak MNI coordinates: −54, 18, −15, regions: the left superior temporal gyrus and the left superior temporal pole. The mean ReHo-values extracted from the peak voxels identified by the ReHo-behavioral correlation analysis were plotted (Figure 6B). Individuals with higher local connectivity in the left ventral anterior insula and the left superior temporal pole showed bigger behavior change in the DG, i.e., they were more likely to be affected by the previous unfair treatment and offered less to unrelated partners.

## Discussion

The goal of the present study was to examine the consequences of unfair treatment in subsequent interactions with new interaction partners and the associated neural mechanisms. The experiment revealed the impact of unfair treatment upon subsequent decision-making. Participants dictated fewer allocations to unrelated partners if they had been treated unfairly in the preceding ultimatum game compared to fair treatment, only when they were first exposed to unfair UG/DG. The activation in the right dorsal anterior insula during unfair offers in UG was positively correlated with the behavior change in DG. In addition, preceding unfair treatment was successful in modulating the neural activity underlying subsequent social decision-making. Specifically, activity in the right dorsal anterior insula was enhanced in the DG block following unfair treatment relative to the DG block following fair treatment. Moreover, the regional synchronization of voxels within the left ventral anterior insula and the left superior temporal pole, as indicated by the ReHo-value, was positively correlated with the behavior change between the DG blocks, clueing on the role of emotional responses and the theory of mind in individual variations in social decision making.

Most participants (63%) showed the behavior change of making more unfair offers following unfair treatment. This effect was modulated by the order of block. When participants were first exposed to fair UG/DG and then exposed to unfair UG/DG, they showed no behavior change between fair DG and unfair DG. Participants who were first exposed to unfair UG/DG and then fair UG/DG made fewer offers in unfair DG than those in fair DG. We suggest that participants who were first exposed to fair UG were preadapted to fair social norms and were less likely to be affected by subsequent unfair treatment. Participants who were hurt by unfair treatment could be recovered by lately fair treatment and thus increased their offers when interacted with unrelated partners. Future research is needed to test the sequence effect and to verify the motivation behind participants' behaviors.

Brain areas associated with punishment, such as striatum, VMPFC, and periaqueductal gray (de Quervain et al., 2004; Strobel et al., 2011; White et al., 2014), did not show differential responses to decision making after unfair treatment and fair treatment. These results lend little support for the hypothesis that the behavior change after unfair treatment was mainly motivated by punishment impulse akin to reactive aggression (White et al., 2014). One possibility is that the current design is not appropriate to test the hypothesis. After all, the task is altruistic decision making rather than punishing, and we did not manipulate punishment *per se*.

Importantly, we found differential activity in the right dorsal anterior insula for the DG trials following unfair treatment vs. following fair treatment, and the activation of the right dorsal anterior insula during unfair offers in UG correlated with behavior change in DG. The anterior insula is active during a wide variety of tasks involving physiological and interoceptive awareness (Craig, 2002, 2009; Critchley et al., 2004), perception, experience, and anticipation of emotion (Lévesque et al., 2003; Nitschke et al., 2006; Duerden et al., 2013), empathizing with others who are in negative emotional states (Singer et al., 2004), and processing information about risk and uncertainty (Preuschoff et al., 2008; Rao et al., 2008; Xue et al., 2010). In social interaction tasks, the anterior insula was activated during aversive emotional experiences associated with strong visceral and somatic sensations, such as facing unfairness (Sanfey et al., 2003; Tabibnia et al., 2008; Kirk et al., 2011), facing the threat of punishment (Spitzer et al., 2007), and having had a promise broken (Baumgartner et al., 2009). The anterior insula has been highlighted as a region that integrates sensory, affective and bodily information with information about uncertainty to generate a dominant subjective feeling state and modulates social and motivational behavior in conjunction with bodily homeostasis (Singer et al., 2009). Recent reviews afford new insights into the functional dissociations within the anterior insula. While the ventral anterior insula network was associated with emotion, chemosensation, and autonomic function, the dorsal anterior insula network was associated with higher cognitive tasks and executive control, such as task switching, inhibition, error processing, feedback evaluation, and conflict detection (Nieuwenhuys, 2011; Chang et al., 2013). In the present study, the right dorsal anterior insula is sensitive to unfair offers, which is consistent with the meta-analysis result of a recent study on the brain and the UG (Gabay et al., 2014). The right dorsal anterior insula was also enhanced in the unfair DG vs. with fair DG during the offer-considering phase. Sanfey et al. (2003) suggested that the anterior insula represents the negative emotional state induced by unfair offers (Sanfey et al., 2003). Recently, researchers argued that the sensitivity of the dorsal anterior insula to unfairness might go beyond representing negative emotions (Gabay et al., 2014), it may also be involved in representing the violation of social norms (Civai et al., 2012), as a previous study with a similar UG design showed that the anterior insula correlated with both positive and negative norm prediction errors (Xiang et al., 2013). Relevant to the current findings, White et al. (2014) found significant overlap in the modulation of activity within dorsal anterior insula by offer unfairness and punishment delivered, and suggested that anterior insula “orchestrates potentially necessary changes in behavioral response” (White et al., 2014, p. 2143). Importantly, while anterior insula activation has been implicated in the experience of unfairness (see Gabay et al., 2014 for a review), and in the punishment of unfairness (White et al., 2014; Wu et al., 2014), here we show that activation in this region was also associated with behavior change during interaction with unrelated others after exposure to the unfair treatment. This finding highlights the role of anterior insula in leading subsequent decision-making in which the anterior insula not only represents fairness violations (in UG), but also accommodates to the social contexts of preceding unfair treatment (in subsequent DG), supporting the hypothesis that the anterior insula is involved in a more general mechanism of behavioral adaption, and modulates subsequent decision making in complex social environments (Lamm and Singer, 2010).

Interestingly, we found that the regional synchronization in the left ventral anterior insula and the left superior temporal pole was positively correlated with participants' behavior change between the DG blocks. Analysis of resting-state functional connectivity has suggested that anterior insula is one of the key nodes in the salience network (Sridharan et al., 2008). It serves to detect salient events and initiates appropriate control signals to regulate behaviors (Menon and Uddin, 2010). The ventral anterior insula is predominantly engaged in internal and bodily homeostatic regulation, and is activated by personal emotional and social emotional tasks (Lamm and Singer, 2010; Nieuwenhuys, 2011; Chang et al., 2013). Individuals with increased local connectivity in the left ventral anterior insula might be more sensitive to environmental arousal information, and thus might be more likely to be influenced by preceding unfair treatment and showed larger behavior change in the DG blocks.

The temporal pole is the most rostral portion of the temporal lobe and has been implicated in different cognitive functions such as emotion, attention, behavior, and memory (Blaizot et al., 2010). However, it is primarily considered a key region for theory of mind processing (Bodden et al., 2010; Jimura et al., 2010; Park et al., 2011). Though several distinct brain regions may be activated during theory of mind, forming an integrated functional network, a review of neuroimaging literature suggests that the superior temporal regions are core regions in the network and are associated with theory of mind reasoning in 50% of the studies (Carrington and Bailey, 2009). Participants with stronger local connectivity in the left superior temporal pole showed larger behavior change in the DG blocks. This result is counterintuitive at first sight: how would the theory of mind lead to unfair behavior against neutral partners (dictating less in the DG following unfair treatment)? Previous study showed that children who had acquired theory of mind proposed higher mean offers than did children who had not acquired theory of mind, implying that the ability to infer the mental states of others plays an important role in fairness-related behavior (Takagishi et al., 2010; but see Cowell et al., 2015). However, other studies suggest that a well-developed ability of theory of mind enhances competitive skills and facilitates strategic behaviors as it enables individuals to take advantage of others in order to realize their own goals (Paal and Bereczkei, 2007). A recent electroencephalography study with repeated ultimatum game found that participants with higher score in the social cognition test made lower offers to partners and had greater prefrontal theta activity, suggesting that participants with better ability to read others' intentions tend to behave strategically and expect others to accommodate to their own intentions (Billeke et al., 2014). In the present study, the significant ReHo-behavioral correlation in the left superior temporal pole might implicate that participants with better ability of theory of mind were more likely to show behavior change between the DG blocks. A possible explanation is that participants with increased baseline activity in the superior temporal pole were more likely to use contextual information and to gain advantageous position, and were more frequently to make strategic decisions (e.g., made fewer offers to partners and more benefit for their own following unfair treatment). These results extend those of prior studies that suggest the important role of the theory of mind in fairness-related behavior (Rilling et al., 2004; Takagishi et al., 2010), and confirm the critical role of the temporal pole in representing and retrieving social knowledge (e.g., emotionally tagged knowledge) that is used to guide higher-level social behaviors (Olson et al., 2013). It is worth noting that the finding of a correlation with behavior change in the superior temporal pole does not necessarily indicate that the theory of mind function is impaired in participants who showed little behavior change (anonymous reviewer's suggestion). The present study only provides preliminary evidence that individuals who have stronger local connectivity in the left superior temporal pole are more likely to show adaptive behavior to social contexts.

It should be noted that the DG activation results failed to replicate previous findings from similar studies. Relevant regions such as the DLPFC and the ACC, which have been shown to be involved in fairness-related decision-making (Weiland et al., 2012; White et al., 2014), were not found in the current study. This result could have been attributable to the study design and the data analyses. Unlike previous studies that examined the brain activity of proposers that contributed to fair and unfair decisions (Weiland et al., 2012) or the punishment of unfairness (White et al., 2014), the main focus of the current study was the behavioral and neural differences between decision making following fair and unfair treatment. For example, the DLPFC might have shown enhanced activity when participants were thinking about what type of offer to make in the DG following both fair and unfair treatment. The current results suggest an insensitivity of the DLPFC and the ACC to preceding unfair treatment. Nevertheless, they do not indicate the non-involvement of the DLPFC and the ACC in fairness-related decision making.

Our general finding of unfair transfer to a neutral person after unfair treatment is notable for several reasons. First, the partners with whom our participants interacted in the two DG blocks were not responsible for the previous unfair or fair treatment. In light of this feature, one might have expected the present decision making to depend primarily on self-interest and to show no difference between the blocks, but that was not the case. We emphasize that our use of unrelated partners (rather than related offenders) underscores the present conclusion: even innocent others could be affected by unfair treatment. We also note that our findings are broadly consistent with research on the neural bases of fairness-related decisions (Sanfey et al., 2003; Weiland et al., 2012; Grecucci et al., 2013). Our results suggest that social norm violations, as indexed by the activity in the anterior insula, could impact subsequent decision making.

Two of the limitations of our study are the lack of post-experiment interviews regarding the reasons for participants' decisions and the absence of any theory of mind/empathy measures to support the hypotheses. A further limitation is that the participants were mainly female. However, no gender difference was found in their behavioral responses.

In summary, the present study provides empirical evidence that suffering from unfairness could influence subsequent decision making in interactions with unrelated others. Enhanced activation of the right dorsal anterior insula, a region that is involved in representing social norm violations, was observed during and after unfair treatment. Furthermore, increasing baseline activities in the left ventral anterior insula and the left superior temporal pole were associated with larger behavior change following fair and unfair treatment. These results may represent the role of these regions in adaptive social decision making. The finding of the consequences of unfairness may have a wide range of implications for our understanding of daily social life.

### Conflict of interest statement

The authors declare that the research was conducted in the absence of any commercial or financial relationships that could be construed as a potential conflict of interest.
